# Development and evaluation of an open source Delphi-based software for morphometric quantification of liver fibrosis

**DOI:** 10.1186/1755-1536-3-10

**Published:** 2010-06-17

**Authors:** Sebastian Huss, Jörg Schmitz, Diane Goltz, Hans-Peter Fischer, Reinhard Büttner, Ralf Weiskirchen

**Affiliations:** 1 Institute of Pathology, University of Bonn, Bonn, Germany; 2 Institute of Clinical Chemistry and Pathobiochemistry, RWTH University Hospital Aachen, Germany

## Abstract

**Background:**

Computer-based morphometry can minimize subjectivity in the assessment of liver fibrosis. An image processing program was developed with Delphi for the quantification of fibrosis in liver tissue samples stained with Sirius Red. Bile duct ligated and sham operated wild type C57BL/6 mice served as a model of time-dependent induction of liver fibrosis. Formation of fibrosis was determined with the developed software at day 0, 3, 7, 10, 14, 20, 30 and 60. The results were compared to a semi-quantitative scoring system.

**Results:**

Quantitative accumulation of collagen fibres was observed from day 3 to day 14, with a slight further increase thereafter. During ongoing fibrogenesis, there was a significant elevation of alanine aminotransferase (ALT), aspartate transaminase (AST) and bilirubin. The results from our computer-based morphometric analysis were highly correlated with the results that were obtained in a standardized pathology semi-quantitative scoring system (*R *^2 ^= 0.89, *n *= 38).

**Conclusions:**

Using our Delphi-based image analysing software, the morphometric assessment of fibrosis is as precise as semi-quantitative scoring by an experienced pathologist. This program can be a valuable tool in any kind of experimental or clinical setting for standardized quantitative assessment of fibrosis.

## Background

Liver fibrosis is characterized by an increase of collagenous matrix (the quantitative aspect of fibrosis). There is also a reduction of the vascular bed, pathologic perfusion, atrophy and regeneration of parenchyma leading to a fundamental rebuilding of tissue architecture (qualitative aspect of fibrosis). Complete organ fibrosis represents the final course of chronic progressive liver diseases.

Pathologists describe changes in these two dimensions subjectively or semi-quantitatively by a variety of scoring systems depending on the underlying disease causing different histological patterns. For example, in chronic hepatitis the Ishak score [1] is used. In alcoholic or non alcoholic steatohepatitis, fibrotic progression has been quantified by Brunt and colleagues [2]. Cholangiodestructive and cholangitic diseases are scored according to Portmann and Nakanuma [3]. A computer-based morphometry for the assessment of liver fibrosis is currently not in use for standard clinical diagnostics but is sometimes used scientifically in experimental models [4-8].

The primary goal of the present study was the development of highly user-friendly, charge-free and open source computer software to assess the quantitative aspect of liver fibrosis in a standardized and reproducible manner. Therefore, we used the model of bile duct ligation (BDL) to induct liver fibrosis in C57/BL6 mice. The experimental model has been well described and evaluated in rats and mice [9,10] and has been widely used to study cholestatic liver injury [11,12] and fibrogenesis [9,13,14]. We analysed time-related quantitative and semi-quantitative aspects of murine liver fibrosis and evaluated the different measurement techniques.

## Results

### Impact of bile duct ligation on survival, activity and jaundice

**Figure 1:**
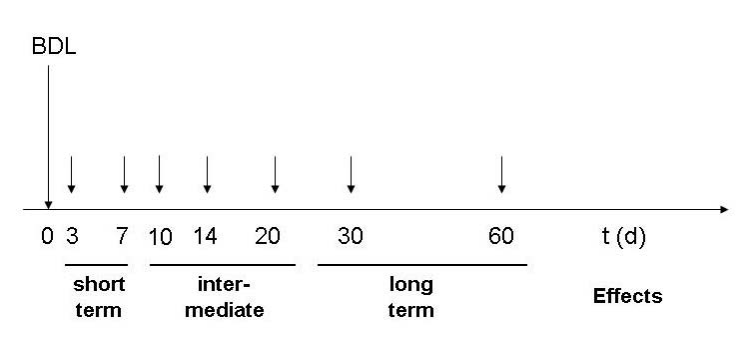
**Study design and experimental setting**. Bile duct ligation was performed on day 0. Analysis was performed on day 3 and 7 after the procedure to measure short time effects. Time-points after 10, 14 and 20 days were chosen to display intermediate and 30 and 60 days to analyse long-term effects.

We have performed BDL in 40 animals and analysed ongoing hepatic fibrogenesis in respective animals at fixed time points (Figure 1). All sham-operated animals survived but two of 40 mice in the BDL group became moribund and were killed before the planned end point. The activity of sham-operated animals was nearly normal 24 h after the lapratomy while the animals subjected to BDL showed reduced activity during the first 72 h but regained normal activity thereafter. Jaundiced skin was already apparent in all animals 24 h after BDL.

### Hepatocellular injury and cholestasis after BDL

**Figure 2:**
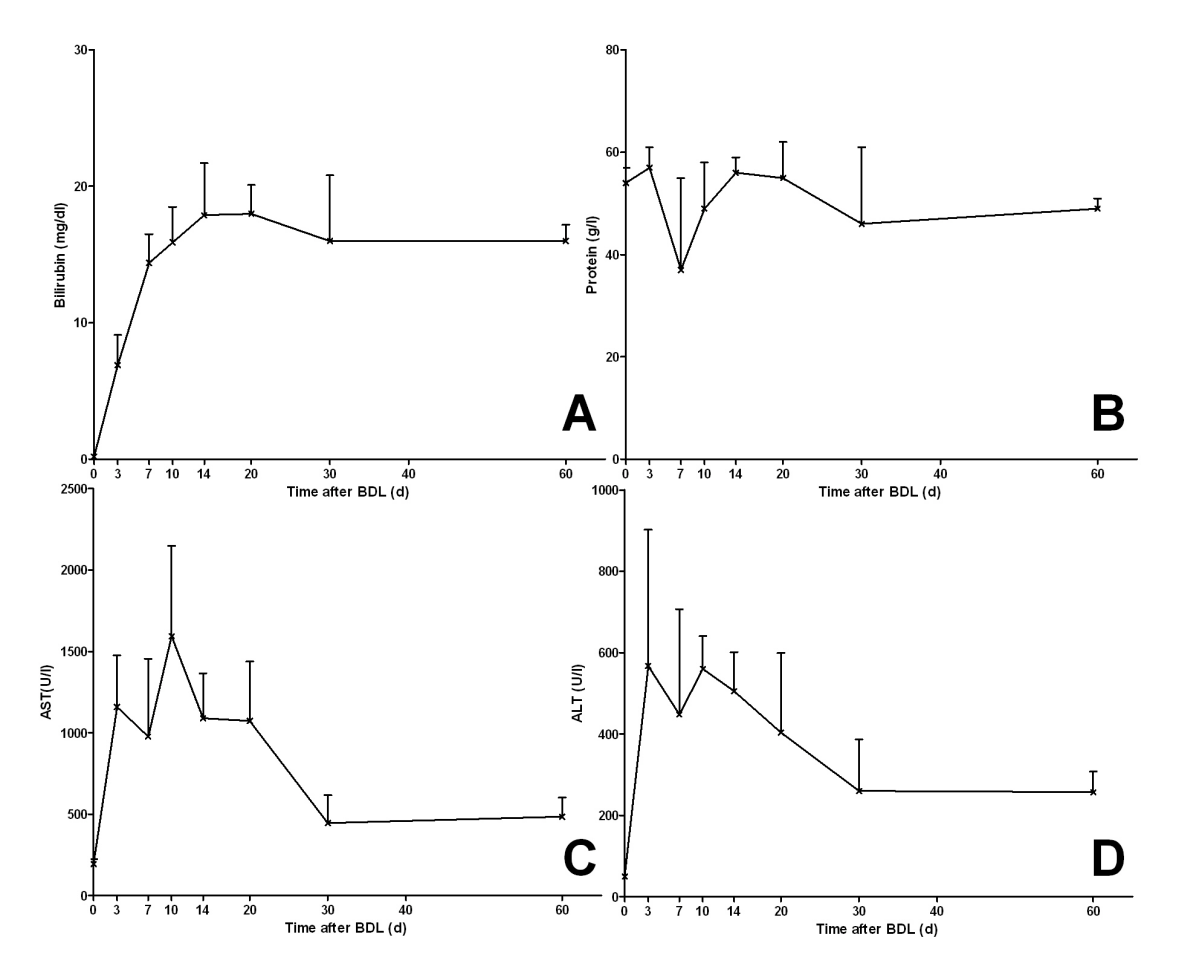
**Serum markers of cholestasis and hepatocellular injury during bile duct ligation**. Biochemical markers are shown for (A) bilirubin, (B) total protein, (C) alanine aminotransferase and (D) aspartate transaminase.

Alanine aminotransferase (ALT) and aspartate transaminase (AST) increased rapidly after BDL, peaking at 7 respectively twenty days after the surgery. After the peaking, ALT and AST decreased steadily until day 30; serum levels remained almost unchanged after 60 days. Bilirubin levels steadily elevated and reached a plateau after 7 days. Total protein serum levels had a greater variability with a slight decrease after 7 days (Figure 2).

### Development of liver fibrosis

**Figure 3:**
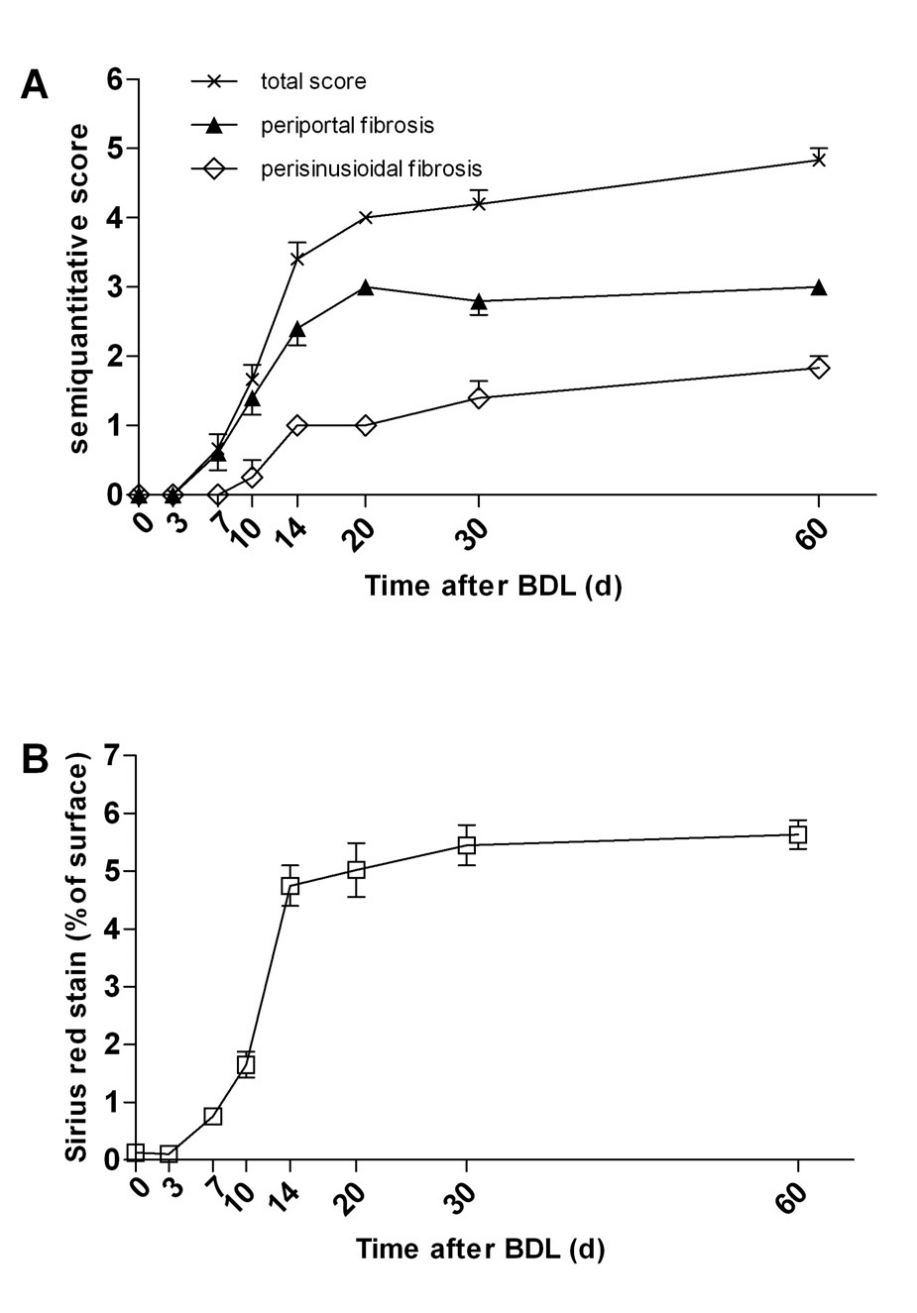
**Semi-quantitative analysis versus computer-based morphometrical analysis**. (A) A semi-quantitative score that considers both periportal and perisinusoidal fibrosis was determined and compared to (B) the morphometric analysis determined in Sirius Red stained specimen.

The stage of fibrosis was assessed semi-quantitatively. Periportal fibrosis was staged 0-4 and perisinusoidal fibrosis was scored 0-2 by an experienced pathologist (HPF), giving a maximum possible of 6 (Table 1). The morphometric computer-based assessment of fibrosis showed 0.13 ± 0.037% fibrosis-index in the sham operated group. A strong accumulation of collagen fibres was observed between day 3 (0.10 ± 0.03%) and 14 (4.75 ± 0.35%), with a slight further increase thereafter (Table 2, Figure 3). The mean fibrosis score in sham operated animals was 0.00 ± 0. It increased steadily until day 60 to 4.83 ± 0.17. The maximum of periportal fibrosis (stage 3; complete lamellae) was reached at day 20 (3.0 ± 0.0). Peripsinusoidal fibrosis was absent during the first 10 days and was established after 14 days (1.0 ± 0.0). It increased steadily until day 60 to 1.8 ± 0.17 (Table 2, Figure 3).

**Table 1:** Semi-quantitative scoring of fibrosis: a two-tired scoring system for histopathological liver changes after murine bile duct ligation

Periportal fibrosis	Score
No fibrosis	0

Scattered periportal and perineoductular fibrosis (incomplete lamellae)	1

Periportal, perineoductular fibrosis (complete lamellae) +/- beginning septa	2

Periportal, perineoductular fibrosis with portal-portal septa *	3

Complete cirrhosis	4

**Perisinusoidal fibrosis**	

No fibrosis	0

Mild fibrosis (fibres in < 50% of the perisinusoidal spaces)	1

Severe fibrosis (fibres in > 50% of the perisinusoidal spaces)	2

*Three or more portal-portal septa have to be found per 10 portal fields.

**Table 2:** Fibrosis index and semi-quantitative score

Time after bile duct ligation (days)	Fibrosis index (%)	Semi-quantitative score		
		
		Portal fibrosis	Perisinusoidal fibrosis	Total score
0 (*n *= 3)	0.13 ± 0.04	0.0 ± 0.0	0.0 ± 0.0	0.0 ± 0.0

3 (*n *= 5)	0.10 ± 0.03	0.0 ± 0.0	0.0 ± 0.0	0.0 ± 0.0

7 (*n *= 5)	0.76 ± 0.11	0.60 ± 0.25	0.0 ± 0.0	0.60 ± 0.25

10 (*n *= 5)	1.65 ± 0.22	1.40 ± 0.25	0.25 ± 0.25	1.67 ± 0.25

14 (*n *= 5)	4.75 ± 0.35	2.4 ± 0.25	1.0 ± 0.0	3.40 ± 0.24

20 (*n *= 4)	5.02 ± 0.47	3.0 ± 0.0	1.0 ± 0.0	4.0 ± 0.0

30 (*n *= 5)	5.45 ± 0.35	2.8 ± 0.2	1.4 ± 0.25	4.20 ± 0.20

60 (*n *= 6)	5.63 ± 0.25	3.0 ± 0.0	1.8 ± 0.17	4.83 ± 0.17

Fibrosis index (% Sirius Red stain) and semi-quantitative score consisting of portal fibrosis score, perisinusoidal score and total score (mean +/- standard error of mean; see Table 1)

**Figure 4:**
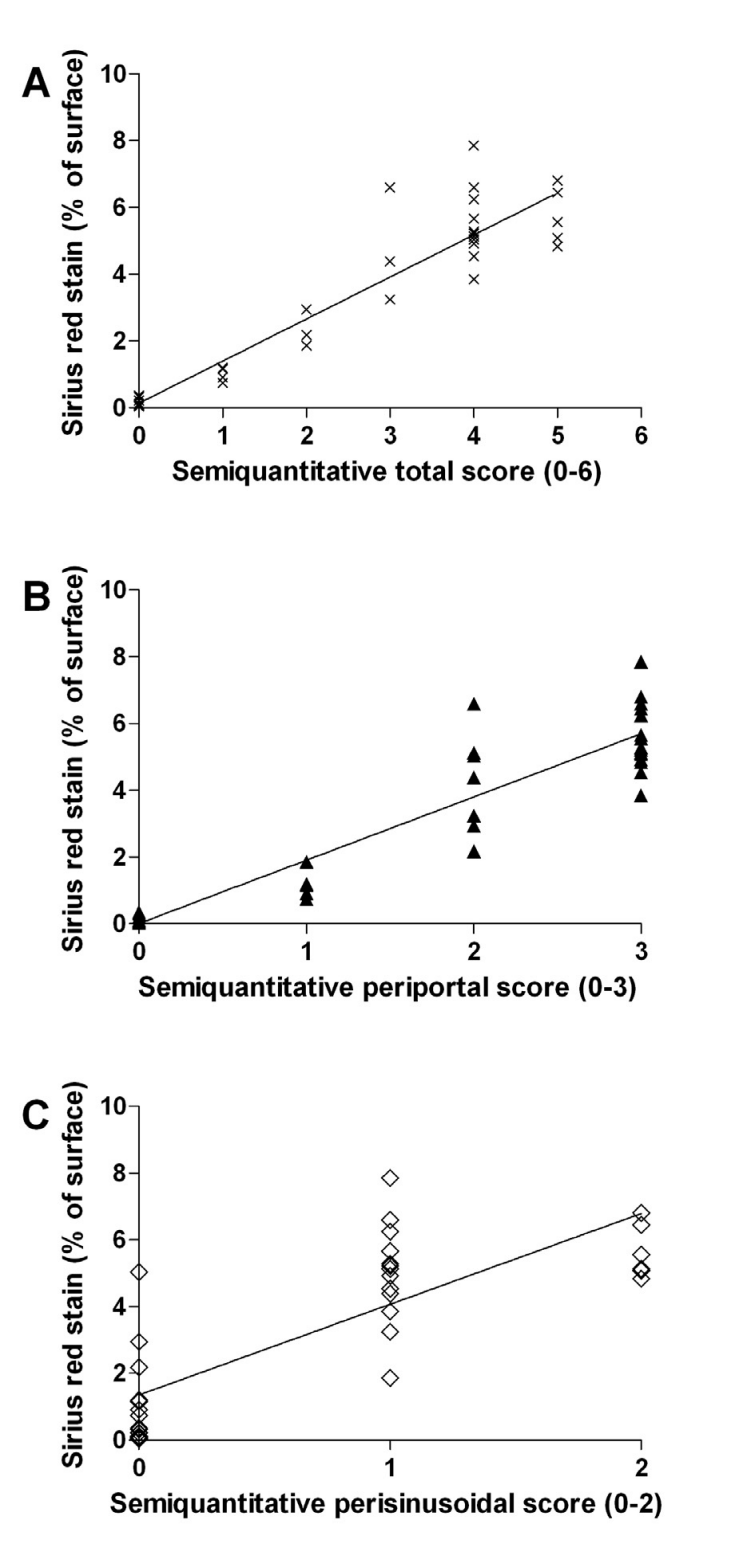
**Correlation between semi-quantitative and computer-based analysis**. Linear regression fit between semi-quantitative total (A; *R*2 = 0.89), periportal (B; *R*2 = 0.86) and perisinuidal (C; *R*2 = 0.64) score and morphometric assessment (Percentage of positively stained surface, Sirius Red Staining; *n *= 38.)

Both the total and periportal semi-quantitative scoring values showed a good correlation with the computer-based assessment and could be fitted to a linear regression curve (*R*^2 ^= 0.89 versus *R*^2 ^= 0.86; *n *= 38). However, semi-quantitative scoring of perisinusoidal fibrosis showed a lower correlation (*R*^2 ^= 0.64, *n *= 38) (Figure 4).

## Discussion

Liver fibrosis is a major parameter guiding the diagnosis and prognosis of chronic liver diseases and liver biopsy and its histological evaluation remains the gold standard for diagnosis and prognosis. Therefore, accurate qualitative and quantitative assessment of fibrosis is essential. Many scoring systems were designed to classify and stage different chronic liver diseases [1,2,15]. One major flaw of these scoring systems is that they are dependent on the visual interpretation of the observer. In addition, the observer must be an experienced pathologist. In order to avoid these pitfalls, over the last decade quantification of fibrosis by multiple computer assisted methods has been introduced [5,7,16,17]. The principle behind these methods is to stain a section with a specific tissue stain that highlights collagen fibres. Then, with the aid of the software, the fibrotic area is calculated. Ideally, the correct assessment of total fibrosis should be possible fully computerized that rules out intra- and inter-observer variations. Dahab *et al*. used the commercial program Adobe Photoshop to calculate a fibrosis index [6]. In addition, there are different companies (Soft Imaging Systems, Münster, Germany or Aperio, CA, USA) who sell programs which have been designed specially for image analysis purpose. These programs are usually highly expensive and are not easily adjustable.

Therefore, we have written a new simple, charge free, computer-based morphometric assessment tool to evaluate liver fibrosis. This software (Fibromat) is written in Delphi - one of the best known and widely used programming tools - to create software and applications for Microsoft Windows computers. With Delphi it is possible to create small powerful applications which do not need to be installed, do not depend on Active X controls or special DLLS. Therefore, there are no problems with installation, as usually observed with software from other programming utilities (for example, Java, .NET, Visual Basic or Visual C++). The program allows the determination of different colour intensities that are necessary when morphometric scoring is necessary, for example, Sirus Red stained specimen. In order to perform a quantitative analysis, respective images of such a stain are first transformed into a grey scale and then further modified into a colour image in which the calculated background is depicted in black, liver parenchyma in light green and fibrous deposits in red. After this conversion, a precise and rapid mathematical quantification (in %) of individual colours is possible.

One major advantage of our program is that it is open source. Therefore, the complete source code of the program is accessibly by downloading it (**→ **Additional file 1) and it is easily adapted it to specific needs. For example one can choose different colors for the morphometrical analysis and make the program suitable for Massons Tricrome Staining or any immunohistochemical staining. With little effort an analysis of different subtypes of collagen is possible.

As previously mentioned, the pattern of fibrosis depends on the aetiology, severity and duration of the underlying disease. We investigated the qualitative and quantitative aspects of murine liver fibrosis caused by bile duct ligation using a semi-quantitative score. The pattern of fibrosis caused by this procedure can be compared, to a certain degree, to the pattern occurring in primary biliary cirrhosis (PBC). There is a type of periportal fibrosis that, along with the proliferation of neoductules and periductular fibrosis, leads to porto-portal septae [18]. A semi-quantitative scoring system for PBC was first described in 1965 by Rubin *et al*. [19]. They described four successive stages, which were modified by others [20-22] and summarized by Portmann and Nakanuma [3]. Other work groups scored their BDL-experiments according to one of these systems [23,24].

However, the scoring systems discussed were originally designed to score PBC, an autoimmune liver disease with an unknown aetiology where selectively small intrahepatic bile ducts are destroyed. The pathogenic mechanism has to be distinguished from that occurring in BDL, where the common bile duct is ligated. Secondary to this procedure, small bile ducts react and form neoductuli [9].

We propose an alternative approach to assess fibrotic changes after murine bile duct ligation with a two-tired scoring system. Modified after Portmann and Nakanuma [3] we staged periportal fibrosis as follows: 0, no fibrosis; stage 1, focal periportal and perineoductular fibrosis that build incomplete lamellae around affected portal fields; stage 2, fully established periportal and perineoductular fibrosis building complete lamellae, with or without sporadic portal-portal bridging; stage 3, an extension of the portal-portal bridging (three or more bridges per 10 portal fields); and stage 4, fully developed cirrhosis (Additional File 2). However, stage 4 was not observed in our experimental setting. This finding is different to descriptions of the rat model. Here fibrosis is progressive and cirrhosis can develop within 15 days after BDL [25]. Furthermore, we staged perisinusoidal fibrosis as follows: 0, no fibrosis; stage 1, a mild fibrosis with less than 50% of the lobule affected; and stage 2, a severe fibrosis with more than 50% of the lobule affected.

In our model, periportal fibrosis was fully developed after 20 days; persinusoidal fibrosis become evident at day 10 and increased until the end of the experiment (day 60). When semi-quantitative scoring data are evaluated, it is important to bear in mind that the numbers represent categories rather than measurements. They, therefore, cannot be used as real numbers in statistical analyses [26,27]. Also adding the numbers representing different grading components (periportal and perisinusoidal) together to create a total score can lead to inaccuracies. At best, a total grading score can give only an approximate idea of the severity of disease [28]. We, therefore, correlated every dimension as well as the total score of our two-tired scoring system with the calculated fibrotic-index. We could show that the periportal fibrosis, as well as total score, is highly correlated but perisinusoidal fibrosis is lower. These findings suggest that a qualitative assessment of the computerized fibrosis pattern might still be necessary for an accurate interpretation of computerized fibrosis ratio, because a merely quantitative fibrotic-index of a specimen does not display all the information supplied by a visual interpretation of the slide. Nevertheless both, the semi-quantitatively scoring and the computer-based assessment (fibrotic-index) showed good correlation in depicting increased collagen deposition as a consequence of ongoing fibrogenesis. Therefore, we propose this highly user-friendly image analysis tool for the accurate quantification of collagen deposits in Sirius Red stained liver sections. The image processing is computerized and, for this reason, more insensitive to intra- and inter-observer variations than a semi-quantitative scoring system.

## Conclusions

We designed an easy-to-use, charge free and open source Delphi-based computer program to assess the quantitative aspect of liver fibrosis in a standardized and reproducible way. The program was evaluated in an experimental setting of murine liver fibrosis following bile duct ligation. It can also be used for the analysis of fibrosis due to other aetiopathologies.

## Methods

### Animals

Male C57BL/6 mice (Harlan Laboratories, Eystrup, Germany) aged 10-12 weeks were kept under controlled environmental conditions with a 12-h light-dark cycle for a minimum of 7 days before surgery. Mice were fed on a standard laboratory diet with food and water *ad libitum*. All experiments were approved by the Landesamt für Natur, Umwelt und Verbraucherschutz NRW, Recklinghausen, Germany (AZ 50.203.2 AC20, 13/06 and AZ 8.87-50.10.37.09.248).

### Procedures

#### BDL

After midline skin lapratomy the liver was gently removed and the common bile duct was mobilized. In the region above the pancreas two 4-0 nylon sutures were placed around the bile duct and carefully tightened to avoid rupture.

#### Sham lapratomy (control)

Lapratomy and mobilization of the common bile duct were performed as in the former group but without ligation.

In order to measure short time effects of the BDL, mice were sacrificed at day 3 and 7 after the surgery. In order to indicate intermediate time effects mice were sacrificed after 14 and 20 days, to assess long term effects after 30 and 60 days. Control animals were killed 20 days after the sham lapratomy. At indicated time points, blood was drawn from the right ventricle, centrifuged and the serum was stored at -80°C until further analysis. The liver was removed and fixed in 4% buffered formalin for 24 h for histological analysis.

### Measurement of serum parameters

Blood biochemical parameters (bilirubin, ALT, AST and total protein) were measured using the Modular Pre-Analytics (MPA) system (Roche Diagnostics, Mannheim, Germany)

### Histology

After being fixed in 4% buffered formalin for 24 h, the liver tissue was embedded into paraffin wax. A histological semi-quantitative examination of the liver was performed on sections after standard Sirius Red staining. Periportal fibrosis was staged 0-4 and perisinusoidal fibrosis was scored 0-2, giving a maximum possible of 6 (Table 1).

### Development of the image analysing program

The principle behind computer-based morphometry is the different staining pattern of cells, nuclei and fibres following Sirius Red staining. Collagen fibres, as well as cell nuclei, appear red, while the hepatocellular cytoplasm becomes pale and yellowish.

**Figure 5:**
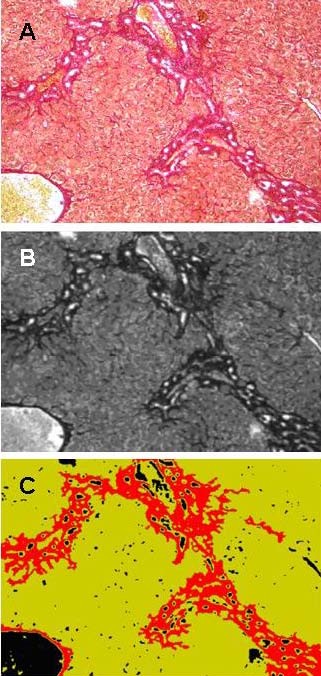
**Computer-based morphometry**. (A) Photograph of a Sirius Red stain of a liver section (100 × magnifications). (B) Grey transformation, blurring and shading of respective photograph. (C) Resulting image of respective photograph. Background is black, liver parenchyma light green and fibrosis intuitively red.

**Figure 6:**
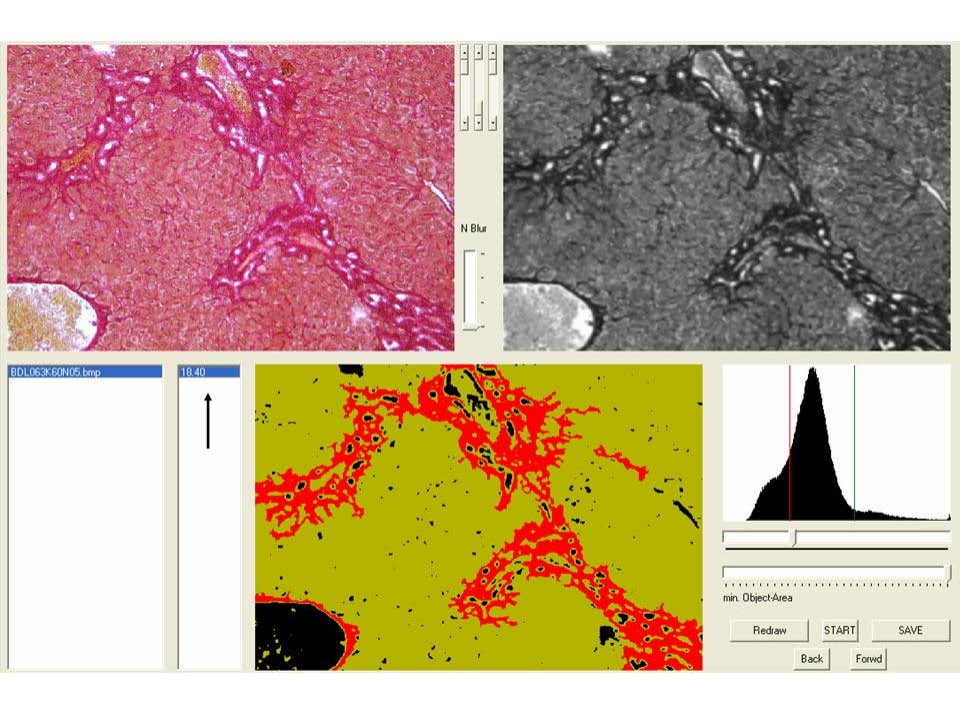
**Screenshot of program interface**. The original photographs are shown in the upper left corner. In the upper right corner the same picture is shown after adding the grey transformation, blurring and shading. The histogram analysis is shown in the lower right corner. The image in the lower middle shows the calculated background in black, the liver parenchyma in a light green and fibrosis intuitively red. The percentage of fibrous tissue is calculated on the lower left side (black arrow).

For the analysis, 10 photographs of random high-power fields (100 × magnifications) were taken of each liver sample. Large bile ducts and vessels were excluded. Photographs were stored as 1280 × 1024 pixel RGB-bitmaps (bmp) with a colour-resolution of 24 bits per pixel. These pictures were analysed with our Delphi-based program as follows. After a shading correction, a grey transformation - derived from the green color channel only - is calculated from the original image. Then a blurring filter based on an arithmetic mean filter is applied. Thresholding of the positively stained collagen fibres is performed by a histogram analysis of the grey value distribution resulting in a binary image. As cell nuclei and fibres appear to have the same staining intensity, binary object detection is performed and all objects with an area lower than a specific threshold are eliminated, distinguishing between fibres and nuclei. The total area of the combined fibres is expressed as a percentage of the total parenchyma area. These steps are performed automatically and the displayed resulting image shows background in black, liver parenchyma in a light green and fibrosis intense red (Figures 5 and 6). Nevertheless, the user has several options to interact with the program, eliminate different common errors and to simplify his work. (i) Sirius Red staining can have different intensities due to staining time and/or thickness of the histological slide. The user can modify the threshold of the grey transformation to minimize these effects. (ii) If random sections of a sample were taken there may be flapped areas or other artifacts giving a wrong signal. The user can exclude obviously mistaken objects by a simple mouse click. All steps of the complete procedure are visualized in one display and are therefore easy to follow. (iii) Lumina of small vessels, as well as the background, are automatically excluded from the total area of the parenchyma. (iv) Unlimited photographs can be analysed one after another. The user can save and reopen the current working file or send it to a co-worker who can proceed or reevaluate the work. (v) The values (0.00-1.00, that is 0% to 100% fibrosis) can be exported to a text file and re-imported into Excel, GraphPad, SPSS or another program for statistical analysis. The software, which is compatible with Microsoft Windows 7, Microsoft Vista SP2, Microsoft Windows XP Home or Professional (SP2 or SP3), can be downloaded from the journal home page (Additional File 1). Details of programming are available on request.

### Statistical analysis

All results are expressed as the mean values ± standard error of mean, except for serum parameters of the animals, which are expressed as mean values ± standard deviations. Semi-quantitative scores were correlated with the values from the computer assisted morphometrical analysis using Graph Pad Prism software.

## Abbreviations

ALT: alanine aminotransferase; AST: aspartate transaminase; BDL: bile duct ligation; bmp: bitmaps; PBC: primary biliary cirrhosis.

## Competing interests

The authors declare that they have no competing interests.

## Authors' contributions

SH did the animal experiments, helped with the development of the program and drafted the manuscript. JS was the principle programmer and performed the quantitative analysis. DG helped with the animal experiments. RB supervised the animal experiments and participated in the study design. HPF performed the histopathological semi-quantitative scoring. RW was responsible for the serum analysis and was involved in the study design as well as in revising the manuscript for important intellectual content. All authors carefully read and approved the final manuscript.

## Supplementary Material

Additional file 1 **The Fibromat, an open source Delphi-based software for computer-based morphometry**. Download the file and unzip it to your hard disc. Open Delphi (installation is required; program can be obtained from Borland, Texas, USA). Open the file 'ihc.exe' and start the program. If you have any questions on how to use or modify the program feel free to contact us via the corresponding author. Click here for file

Additional file 2 **Scoring periportal fibrosis**. (A) A normal portal field without fibrosis (stage 0). (B) Focal periportal and perineoductular fibrosis (incomplete lamellae, stage 1). (C) Fully established periportal and perineoductular fibrosis building complete lamellae, with or without sporadic portal-portal bridging (stage 2). (D) Extension of the portal-portal bridging (three or more bridges per 10 portal fields, stage 3); complete cirrhosis (stage 4) is not shown. Click here for file
