# Supplementary figures and images for: Development and evaluation of an open source Delphi-based software for morphometric quantification of liver fibrosis
Source: Fibrogenesis Tissue Repair. 2010 Jun 17;3:10. doi: 10.1186/1755-1536-3-10 (PMC2903497; doi:10.1186/1755-1536-3-10)

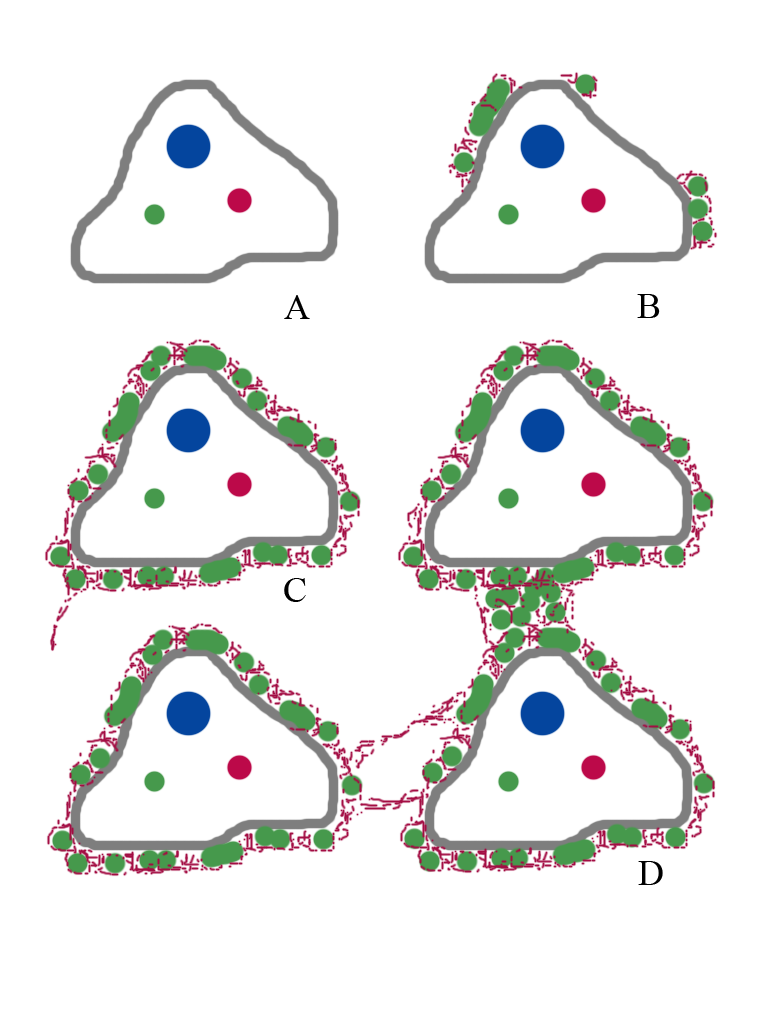

Supplement: Additional file 2 — Scoring periportal fibrosis. (A) A normal portal field without fibrosis (stage 0). (B) Focal periportal and perineoductular fibrosis (incomplete lamellae, stage 1). (C) Fully established periportal and perineoductular fibrosis building complete lamellae, with or without sporadic portal-portal bridging (stage 2). (D) Extension of the portal-portal bridging (three or more bridges per 10 portal fields, stage 3); complete cirrhosis (stage 4) is not shown. [file 1755-1536-3-10-S2.PNG]
